# Inferring the temporal evolution of synaptic weights from dynamic functional connectivity

**DOI:** 10.1186/s40708-022-00178-0

**Published:** 2022-12-08

**Authors:** Marco Celotto, Stefan Lemke, Stefano Panzeri

**Affiliations:** 1 Department of Excellence for Neural Information Processing, Center for Molecular Neurobiology (ZMNH), University Medical Center Hamburg-Eppendorf (UKE), Hamburg, Germany; 2 Neural Computation Laboratory, Istituto Italiano di Tecnologia, Rovereto, Italy; 3 Department of Pharmacy and Biotechnology, University of Bologna, Bologna, Italy; 4 Department of Cell Biology and Physiology, University of North Carolina, Chapel Hill, USA

**Keywords:** Dynamic functional connectivity, Spiking neural network, Communication delay, Transfer entropy, Cross-covariance

## Abstract

How to capture the temporal evolution of synaptic weights from measures of dynamic functional connectivity between the activity of different simultaneously recorded neurons is an important and open problem in systems neuroscience. Here, we report methodological progress to address this issue. We first simulated recurrent neural network models of spiking neurons with spike timing-dependent plasticity mechanisms that generate time-varying synaptic and functional coupling. We then used these simulations to test analytical approaches that infer fixed and time-varying properties of synaptic connectivity from directed functional connectivity measures, such as cross-covariance and transfer entropy. We found that, while both cross-covariance and transfer entropy provide robust estimates of which synapses are present in the network and their communication delays, dynamic functional connectivity measured via cross-covariance better captures the evolution of synaptic weights over time. We also established how measures of information transmission delays from static functional connectivity computed over long recording periods (i.e., several hours) can improve shorter time-scale estimates of the temporal evolution of synaptic weights from dynamic functional connectivity. These results provide useful information about how to accurately estimate the temporal variation of synaptic strength from spiking activity measures.

## Introduction

Neurons in biological networks are sparsely connected by directed, plastic synapses, with communication delays that can vary across different pairs of cells [1–3]. The patterns of synaptic connectivity have a profound influence on the computations and functions of neural circuits [4–6]. Importantly, such synaptic connectivity is not static. The strength of each synapse can change over different time scales—ranging from milliseconds to days—due to processes including synaptic potentiation and depression [7]. Such changes in synaptic weights are thought to be neural-activity dependent and driven by local Hebbian mechanisms of plasticity such as spike timing-dependent plasticity (STDP). In these mechanisms, the potentiation and depression of synaptic weights depends on the precise temporal relationship between pre- and post-synaptic spikes [8].

It is challenging to directly measure time changes of synaptic weights in vivo. One possible approach to study in vivo changes in synaptic strength is to simultaneously record the spiking activity of several neurons within a network and estimate changes in their functional connectivity with the statistical analysis of simultaneous recordings. Though the relationship between fixed structural connectivity and “static” time-averaged functional connectivity (FC), in which FC is computed over long time intervals, has been studied extensively [9–11], how changes in synaptic and functional connectivity relate at different time scales remains unclear.

Understanding the relationship between changes in synaptic and functional connectivity is relevant to a range of neuroscientific questions, such as the role of sleep in synaptic homeostasis and memory formation. Several theories and experimental findings posit that non-REM sleep is accompanied by profound changes in anatomical synaptic connectivity, including the general down-scaling of synaptic connectivity related to homeostasis [12–14] as well as context-specific upscaling in synaptic connectivity, such as sleep-dependent dendritic spine formation after motor learning [15]. The anatomical and theoretical evidence for changes in synaptic strength in sleep have been accompanied by evidence for changes in FC, as observed across the motor network during motor learning [16, 17]. It remains challenging to relate the evidence for structural and functional changes during sleep [18, 19], as robust methods to relate dynamic functional connectivity (DFC) to the underlying temporal evolution of synaptic connectivity are not yet established.

Neural network models are a powerful tool to relate structural and functional connectivity, as the former is known because it is put into the model’s equation by the modeler, and the latter can be computed by activity generated by the model [9, 20]. Previous studies have utilized network models of Izhikevich neurons [1] to investigate the relationship between FC measures and synaptic connectivity because these models are generated by simple equations that can produce firing patterns resembling several types of cortical neurons in vivo [21, 22]. These studies highlighted that static bivariate FC measures, such as cross-covariance and transfer entropy, provide robust estimates of the underlying fixed structural synaptic connectivity in simulated networks. However, they did not examine the temporal evolution of functional and synaptic connectivity within spiking networks incorporating STDP.

Here, we relate the temporal evolution of synaptic connectivity to DFC in a neural network model. We examined the performance of several different DFC methods in estimating the temporal dynamics of synaptic weights (termed dynamic synaptic connectivity or DSC) from up to 180 min of spiking activity in simulated spiking networks whose synaptic strength changed over time due to STDP. We first determined the performance of static FC measures in inferring fixed structural properties of the simulated networks (such as presence or absence of pairwise synaptic connectivity and the associated communication delays). We then applied these measures with a sliding time window approach to compute DFC and quantify its relationship with DSC. We found cross-covariance outperformed other DFC measures in capturing the evolution of synaptic weights over time. We also established how to use the information obtained from the static, time-averaged analysis of the network, to enhance the estimate of DSC from DFC.

Part of this work has been presented at the 15th International Conference of Brain Informatics and published as a conference paper [23].

## Simulated spiking network and inference pipeline

**Fig. 1:**
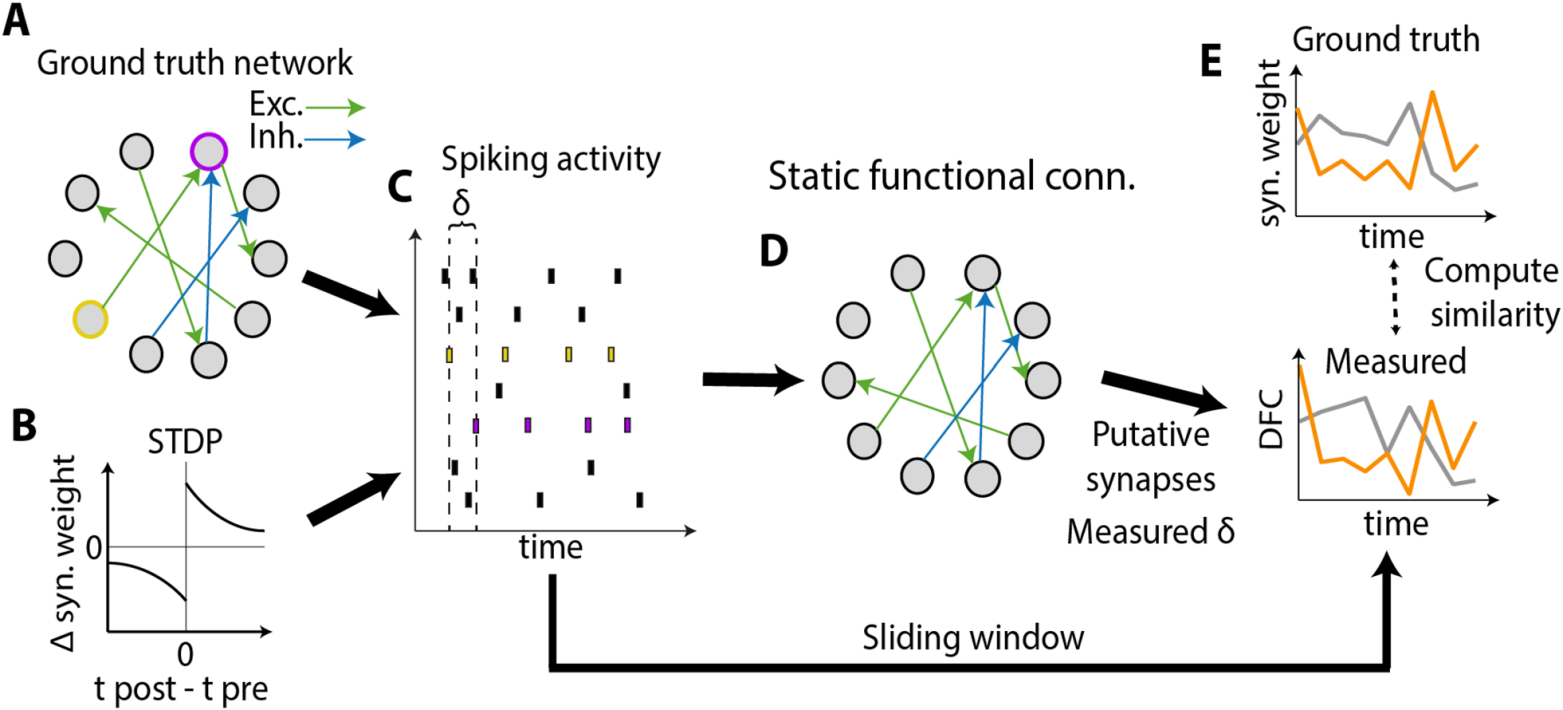
Graphical depiction of the method. **A** Structural connectivity of the simulated network for *N* = 10 neurons. Synaptic weights could be either excitatory (green) or inhibitory (blue). Excitatory connections had randomly distributed communication delays. **B** The strength of the synaptic weights changed over time due to STDP. **C** Structural and biophysical properties of the network determined the spiking activity of the neural population. The presence of an excitatory (inhibitory) synapse between pairs of neurons (such as the yellow and the purple one) generates spike trains that are correlated (anticorrelated) over time with a delay $\delta$. **D** Static FC was measured from spiking activity. **E** DFC was measured from pairwise activity measures, additionaly leveraging on the inferred static connectivity of the network, and compared to ground truth temporal evolution of synaptic weights (two example synapses are shown as orange and grey lines)

To investigate the relationship between DSC and DFC, we simulated a sparsely connected recurrent spiking neural network with heterogeneous synaptic delays across pairs of neurons (Fig. 1a) and synaptic weights evolving over time according to an STDP rule (Fig. 1b). From the simulated spiking activity (Fig. 1c) we computed different FC measures. We then investigated the extent to which these FC measures can be used to infer the “ground truth” synaptic structural connectivity of the network. Namely, we attempted to infer which pairs of neurons were connected, the values of their communication delay, and which synapses were inhibitory or excitatory (Fig. 1d). Then, we used a sliding window to measure DFC and computed the correlation between DSC and DFC over time (Fig. 1e). In doing so, we also studied how exploiting the communication delays estimated via static FC measures could enhance the performance of DFC measures in recovering the ground-truth dynamics of synaptic weights.

We simulated a spiking network of *N* = 100 neurons in which the dynamics of each neuron was governed by the Izhikevich neuron model [24]. Izhikevich derived a single-neuron model which produces a wide set of dynamics that are observed in real spiking neurons, while keeping the computational complexity as low as possible. In this model, the voltage $v$ of each neuron is described by two coupled differential equations:

$$\begin{aligned} & v^{\prime} = 0.04v^{2} + 5v + 140 - u + I_{{{\text{syn}}}} , \\ & u^{\prime} = a\left( {bv - u} \right), \\ & {\text{if}}\quad v\left( t \right) = 30\,{\text{mV}}\quad {\text{then}}\quad v \leftarrow c\quad {\text{and}}\quad u \leftarrow u + d, \\ \end{aligned}$$

where $u$ is a recovery variable, prime symbols (′) denote time derivatives, $I_{{{\text{syn}}}}$ is the total synaptic input to the neuron and $\left( {a,b,c,d} \right)$ is a set of parameters controlling the firing behavior. Depending on the set of parameters, the Izhikevich model can reproduce several firing patterns observed in cortical neurons. As in the original Izhikevich cortical network model [1], we set $\left( {a, b, c, d} \right) = \left( {0.02, 0.2, - 65, 8} \right)$ to simulate excitatory regular spiking neurons, and $\left( {a, b, c, d} \right) = \left( {0.1, 0.2, - 65, 2} \right)$ for inhibitory fast spiking neurons. The term $I_{{{\text{syn}}}}$ is a sum of the voltages generated by the firing of the presynaptic neurons plus an external input term. The external input term consisted of a voltage of 20 mV added to a randomly selected neuron in each simulation time step, as in Ref. [1]. The synaptic voltages were set to an initial value of 6 mV for excitatory synapses and − 5 mV for inhibitory synapses, as in Ref. [1].

**Fig. 2:**
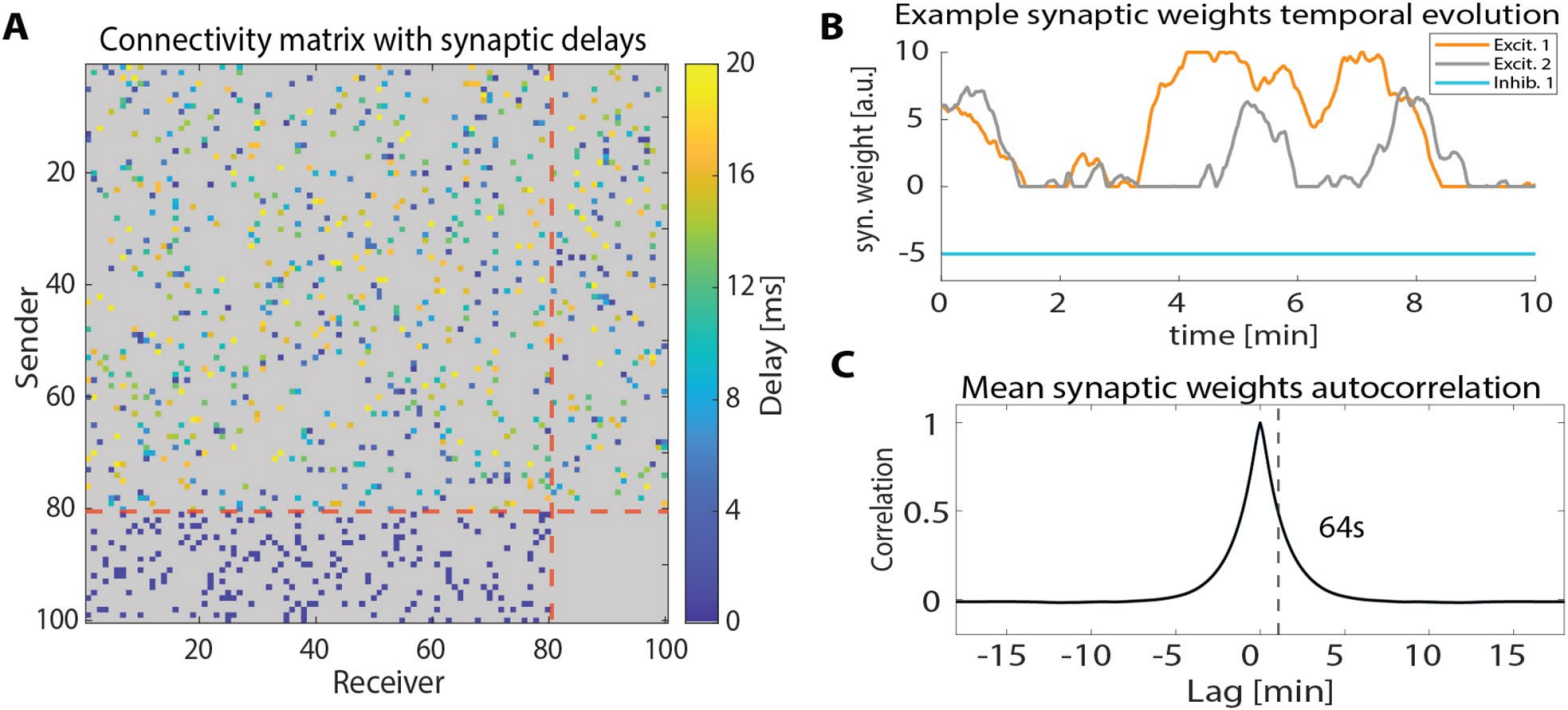
Ground truth connectivity and synaptic weights evolution. **A** Example connectivity matrix, all synapses present in the network were colored according to their communication delay. Absent synapses are grey. The red dashed lines separate the excitatory (*N* = 80 neurons) from the inhibitory (*N* = 20 neurons) population. **B** Ground-truth values of three example synaptic weights over time. Excitatory synapses (in orange and grey) evolved over time due to STDP, inhibitory synapses (in light blue) were constant over time. **C** Autocorrelation function of synaptic weights, the vertical dashed line indicates the autocorrelation half-life (64 s)

As in the original Izhikevich study [1], to match typical proportions of excitatory and inhibitory neurons found in cortex, we set 80% of neurons in the network model to be excitatory and 20% to be inhibitory. Each of the 80 excitatory neurons was randomly connected to 10 excitatory or inhibitory post-synaptic neurons (800 excitatory synapses in total). Each excitatory synapse had a random communication delay whose value was uniformly distributed between 1 and 20 ms and was constant over time. The 20 inhibitory neurons were randomly connected to 10 post-synaptic excitatory neurons (200 inhibitory synapses) with a fixed communication delay of 1 ms. No inhibitory-to-inhibitory (I–I) connections were present in the network (Fig. 2a). The lack of I–I synapses caused the average firing rate of excitatory neurons (5.12 ± 0.08 Hz) to be lower than the one of inhibitory neurons (8.23 ± 0.05 Hz). The simulations ran with 1-ms temporal precision for up to 180 min.

During each simulation, the strength of excitatory synapses changed dynamically (Fig. 2b) due to an asymmetric Hebbian exponential STDP rule: when a presynaptic neuron $i$ fired $\Delta t$ ms before a post-synaptic neuron $j$ the strength of the synapse from $i$ to $j$ ($w_{ij}$) was strengthened as $\Delta w_{ij} = A_{ + } e^{{\frac{\Delta t}{\tau }}}$, on the other hand when $j$ fired before $i$ $w_{ij}$ was depressed as $\Delta w_{ij} = - A_{ - } e^{{\frac{\Delta t}{\tau }}}$ (Fig. 1b). The decay time of the STDP rule was $\tau = 20\,{\text{ms}}$, while $A_{ + } = 0.1$ and $A_{ - } = 0.12$.

Every 1-s synaptic weights were updated by adding $\Delta w_{ij}$ to $w_{ij} .$ After the weights update, the $\Delta w_{ij}$ were not set to zero, but they were multiplied by a memory factor equal to 0.9 and kept as a starting value for the next update. The presence of the memory factor made the synaptic weights evolve over the time-scale of a few minutes (Fig. 2c, autocorrelation half-life = 64 s). To keep the activity of the network balanced, synaptic strengths could not grow above a cut-off value of 10 mV.

## Measures of static and dynamic functional connectivity

**Fig. 3:**
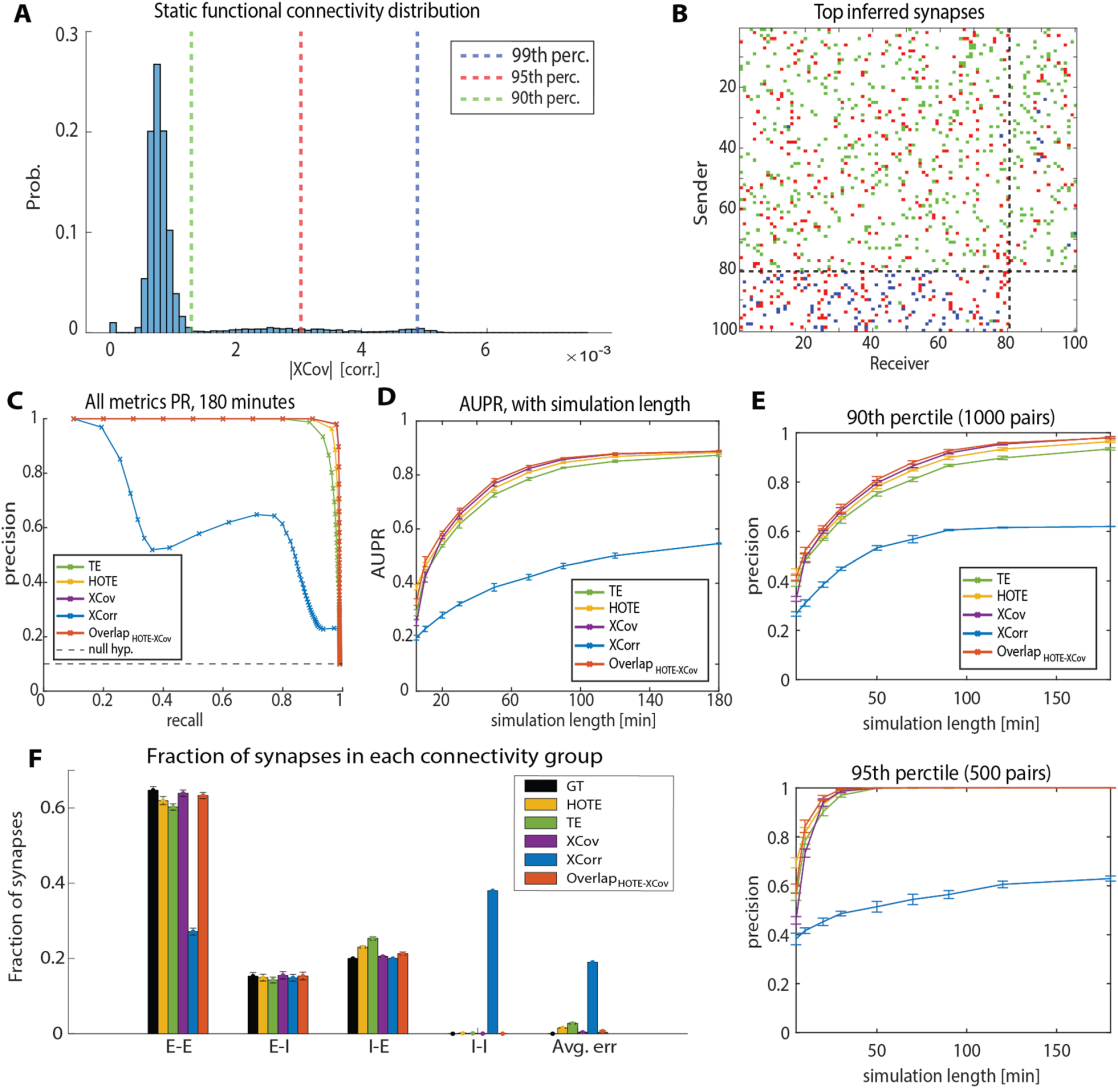
Performance of static FC measures in inferring the presence of synapses. **A** Example distribution of static FC values measured using XCov. The 90th, 95th and 99th percentile were highlighted by dashed vertical lines (in green, red and blue, respectively). **B** Connectivity matrix obtained by considering only synaptic weights above a given percentile of the static FC distribution. The color of synapses represents the percentile (matched with panel A) for which they were included in the connectivity matrix. **C** Precision–recall (PR) curves computed from 180 min of simulated activity for TE, HOTE, XCov and XCorr. Each point is one percentile of the distribution of static FC values across pairs, going from the 1st (bottom right) to the 99th (top left) percentile. **D** AUPR trend with simulation length (length ranges from 5 to 180 min). **E** Comparison of precision in identifying connected pairs with simulation lengths, with a 90th (1000 pairs) and 95th (500 pairs) percentile threshold on the static FC distributions. **F** Fraction of pairs belonging to each group of synapses, from 180 min simulation and with a 90th percentile threshold on the FC distributions GT denotes ground truth. All error bars in the figure are SEM across repetitions of the simulation (*N* = 5)

We used different measures, described below, to compute the static and dynamic functional connectivity of the network from the spiking activity. Such measures were all directed (i.e., could be distinct for each direction between a pair of neurons) and were computed for different temporal delays (*δ*) between the activities of the neurons in the directed pair. When computing static FC, we used data from the whole simulated recording to compute a single connectivity value for each pair of neurons $\left( {i, j} \right)$. We computed all connectivity measures with δ ranging from 1 to 50 ms then, for each pair, we determined the static FC value, denoted as $f_{ij}$, as the maximum connectivity value across delays. We selected the inferred communication delay, denoted as ${\updelta }_{ij}$, as the lag that maximized static FC. After computing $f_{ij}$ for each pair of neurons, we inferred the synaptic connectivity by considering as synaptically connected those directed pairs of neurons whose value of static FC exceeded a threshold value expressed as a given percentile of the distribution of FC values computed across the entire set of pairs of neurons in the network (Fig. 3a, b), as done in previous work inferring the presence of synaptic connectivity from static FC measures [21, 22, 25]. We repeated this procedure separately for each considered measure of FC. When the FC measure was signed, we also inferred whether a synapse was excitatory or inhibitory from the sign of FC. Finally, we used a sliding window approach to compute DFC of all the neurons pairs whose static FC value was in the top 5th percentile of the FC distribution.

Two of the FC measures that we computed were based on Pearson correlation, which is commonly used to estimate the connectivity between pairs of neurons [16, 21, 26]. The first method was normalized cross-correlation (*XCorr*):

$$XCorr_{ij} \left( \delta \right) = \frac{{E\left[ {i_{t - \delta } j_{t} } \right]}}{{\sigma_{i} \sigma_{j} }},$$

where $i_{t}$ and $j_{t^{\prime}}$ are the binary values of the spike trains from neurons $i$ and $j$ at times $t$ and $t^{\prime}$, and the expected value was computed across time. $\sigma_{i}$ and $\sigma_{j}$ are standard deviations of the spike trains of neurons $i$ and $j$, respectively. The second method was the normalized cross-covariance (*XCov*), which subtracts the average firing rate from the spike trains before computing the correlation:

$$XCov_{ij} \left( \delta \right) = \frac{{E\left[ {\left( {i_{t - \delta } - \overline{i}} \right)\left( {j_{t} - \overline{j}} \right)} \right]}}{{\sigma_{i} \sigma_{j} }},$$

where $\overline{i}$ and $\overline{j}$ are the average firing rates of neurons $i$ and $j$, respectively. Given the signed nature of the *XCorr* and *XCov* measures, we first took the absolute value of the measured $f_{ij}$ and then we used the percentiles of this distribution to set a threshold (Fig. 3a, b) to infer whether a synapse was present in the network, regardless of whether it was excitatory (positive correlation) or inhibitory (negative correlation).

We computed two additional FC measures that were variants of the information-theoretic measure of information transfer known as transfer entropy (shortened to *TE*) [27, 28], a measure that has been successfully used to characterize time-dependent changes in recurrent connectivity between mass signals [29]. *TE* has the theoretical advantage of capturing higher-order non-linear interactions as it is defined in terms of the full probability of the lagged activity of neuron $i$ and $j$ and not by lower order features such as correlation values. Additionally, this measure incorporates the Wiener–Granger causality principle of causal communication by conditioning the information between the past of the sender and the present of the receiver neuron on the past activity of the receiver neuron. Our first implementation of transfer entropy uses single time-points statistics to build the probability distribution of lagged neural activity. In mathematical terms, *TE* is defined as follows:

$$TE_{ij} \left( {\updelta } \right){ } = \mathop \sum \limits_{{}} p\left( {i_{t - \delta } ,j_{t} ,j_{t - 1} } \right)\log_{2} \frac{{p\left( {j_{t} |i_{t - \delta } ,j_{t - 1} } \right)}}{{p\left( {j_{t} |j_{t - 1} } \right)}},$$

where $p\left( {i_{t - \delta } , j_{t} , j_{t - 1} } \right)$ is the joint probability distribution of the present state of the receiver neuron $j_{t}$, its past lagged by one time step $j_{t - 1}$ and the past state of the sender neuron lagged by δ time steps $i_{t - \delta }$. The sum occurs over all the $\left( {i_{t - \delta } , j_{t} , j_{t - 1} } \right)$ triplets of events in the probability space. The probability distribution was sampled across time. The lag of the receiver past was set to − 1 ms as using short lags can improve the estimation of the real communication delay [30].

The second implementation of transfer entropy uses multidimensional pasts of the sender and the receiver neuron to consider the possible relevance of time windows longer than 1 ms when transmitting information. Using the terminology of [21], we refer to this measure as higher order transfer entropy (*HOTE*):

$$HOTE_{ij} \left( {\updelta } \right){ } = \mathop \sum \limits_{{}} p\left( {i_{t - \delta}^{\left( k \right)} ,j_{t} ,j_{t - 1}^{\left( l \right)} } \right)\log_{2} \frac{{p\left( {j_{t} |i_{t - \delta}^{\left( k \right)} ,j_{t - 1}^{\left( l \right)} } \right)}}{{p\left( {j_{t} |j_{t - 1}^{\left( l \right)} } \right)}},$$

where $k$ and $l$ are the dimensions of the past activity of the sender and the receiver neurons $i$ and $j$, respectively. For the analysis reported in this paper, we set $k = l = 5\,{\text{ms}}$.

Additionally to the above FC measures, which we already presented in our conference presentation about this topic [23], for the static measures of connectivity we also tested how the overlap between pairs of measures performed in inferring the presence of synapses. We defined the overlap index $O_{ij}^{{\left( {M_{1} ,M_{2} } \right)}}$ between the pair of FC measures $M_{1}$ and $M_{2}$ as:

$$O_{ij}^{{\left( {M_{1} ,M_{2} } \right)}} = \frac{1}{2}\left( {rank\left( {f_{ij}^{{M_{1} }} } \right) + rank\left( {f_{ij}^{{M_{2} }} } \right)} \right),$$

where $rank\left( {f_{ij}^{M} } \right)$ is the rank, in ascending order, of static FC value $f_{ij}$ computed using measure $M$. The sorting of $f_{ij}$ across pairs of neurons was done separately for each measure after maximizing the considered static FC over delays. Therefore, $O_{ij}^{{\left( {M_{1} ,M_{2} } \right)}}$ was used only to infer the presence of synapses and not their synaptic delay ${\updelta }_{ij}$.

## Inferring the presence of synapses from static functional connectivity

We first considered how to infer whether a pair of neurons was synaptically connected. In our simulations, we assumed that neurons were either connected or not connected during the entire simulation, although the strength of their synapse could vary due to plasticity. We assume that the same would apply to data that we analyze with our FC measures.

We computed the FC measures discussed in the previous section between all pairs of neurons and estimated the communication delay for each pair, as explained above. We inferred which pairs of neurons were connected based on a threshold of static FC equal to a given percentile of the distribution of static FC values across all pairs in the network, such that increasing the threshold produced sparser networks (Fig. 3a). A depiction of this is presented in Fig. 3b, where the additional synapses included in the network by lowering the FC threshold are shown in different colors (blue, red, and green for the 99th, 95th, and 90th percentile, respectively). The network obtained including all pairs of neurons whose FC values was above the 90th percentile (i.e., blue, plus red, plus green in Fig. 3b) closely matches the ground truth connectivity matrix (Fig. 2a). To evaluate the performance of different metrics in determining the presence or absence of synapses between pairs of neurons, we compared ground truth connectivity to the connectivity of different inferred networks with static FC thresholds ranging from 1 to 99%. Since the two classes of present and absent synapses were unbalanced (only 10% of all the possible synapses were present in the network), we used precision–recall (PR) curves to study the performance in this classification task [31] (Fig. 3c). Calling $TP$, $FP$ and $FN$ the number of true positive, false positive and false negative inferred synapses, respectively, we have that ${\text{precision}} = \frac{TP}{{TP + FP}}$ and ${\text{recall}} = \frac{TP}{{TP + FN}}$. In other words, precision is the percentage of synapses inferred by the algorithm that are actually present in the network, while recall is the percentage of ground truth synapses that the algorithm correctly identified. Therefore, if for a given measure the two distributions of present and absent synapses were perfectly separable, we would get an optimal PR curve that achieves at the same time ${\text{recall}} = 1$ and ${\text{precision}} = 1$. A random classifier would always have a precision equal to the ratio of synapses present in the model (10%, dashed line in Fig. 3c) for any recall value.

We ran 5 repetitions of a 180-min simulation of the network model, where the identity of synapses present in the network and their communication delay was independently drawn in each repetition. After 180 min, *XCov*, *TE* and *HOTE* all performed well in the classification task, having a PR curve whose shape approached the optimal one which achieves both precision and recall equal to one. Among these three measures, *XCov* showed the best PR curve and *TE* the worst one. The overlap between *XCov* and *HOTE*, denoted as $O^{{\left( {XCov,HOTE} \right)}}$, provided results similar to *XCov*. *XCorr* performed poorly, with a PR curve far from optimal.

The area under the precision–recall curve (AUPR) is a useful metric to summarize the goodness of a PR curve; a perfect classifier has an AUPR equal to one, whereas in our case a random value of AUPR would be 0.1. We compared how the performance of different measures, computed by AUPR, scaled with simulation length. This analysis confirmed that *XCov*, *HOTE* and $O^{{\left( {XCov,HOTE} \right)}}$ were the best metrics in evaluating which synapses were present for long recordings (Fig. 3d). We measured how the precision of the different measures scaled with the simulation time when setting a threshold to the 90th and the 95th percentile of the static FC distribution. With a threshold to the 90th percentile (i.e., 1000 inferred synapses, which equals the ground truth number of connections) we found that the maximum precision in the classification was obtained with *XCov* and $O^{{\left( {XCov,HOTE} \right)}}$, which topped at $98\%$ for 180 min of simulated recording (Fig. 3e, top). With a more conservative threshold to the 95th percentile of connections (i.e., half of the true total number), $O^{{\left( {XCov,HOTE} \right)}}$ captured the top 500 real connections after 30 min of simulation (Fig. 3e, bottom). In general, $O^{{\left( {XCov,HOTE} \right)}}$ had the best AUPR and precision in inferring which synapses were present in the network for simulation lengths ranging from 10 to 70 min. This shows that gathering the information from several measures can boost the inference of fixed structural properties of the network in presence of limited amount of data. To investigate why *XCorr* performed poorly when compared to other measures, we computed the fraction of synapses inferred by each FC measure in the four subgroups of excitatory-to-excitatory (E–E), excitatory-to-inhibitory (E–I), inhibitory-to-excitatory (I–E) and inhibitory-to-inhibitory (I–I) synapses (Fig. 3f). After 180 min of simulated activity and with a 90th percentile threshold on the static FC distribution, *XCov* and $O^{{\left( {XCov,HOTE} \right)}}$ performed best in determining the correct fraction of synapses belonging to each group, while *XCorr* overestimated the number of I–I connections and underestimated the number of E–E connections. This behavior of *XCorr* is due to the differences in average firing rate between inhibitory and excitatory neurons, with a higher firing rate for inhibitory neurons, as *XCorr* is sensitive to the correlation between average firing rates. Given the poor performance of *XCorr* in estimating the presence of synapses, we discarded it in the following analyses.

## Inferring synapse type and communication delay from static functional connectivity

We next studied how well static FC measures performed in inferring whether each synapse was excitatory or inhibitory, and in inferring the value of the communication delay of that pair of neurons. In our model, the communication delay was a fixed structural parameter of synapses across the entire simulation, and we assume that the same holds for data analyzed with our procedure.

**Fig. 4:**
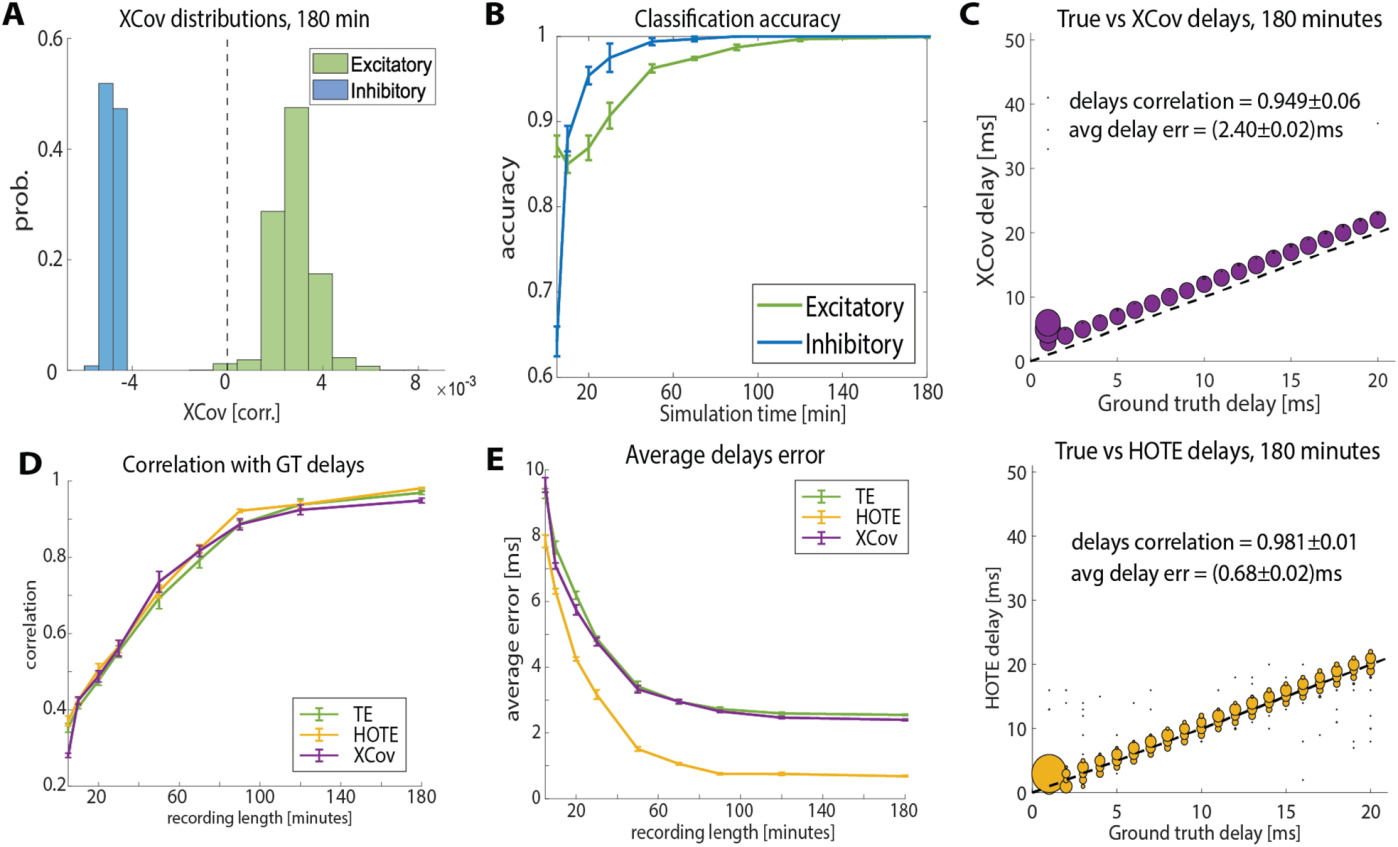
Performance of the measures in estimating connection type and delays. **A** Distributions of static FC values measured using XCov for excitatory (green) and inhibitory (blue) cells. **B** Performance of a classifier in identifying excitatory and inhibitory synapses with simulation length. The decision boundary of the classifier was set to XCov = 0. **C** Scatter plots of real and estimated delays across cell pairs using XCov (top) and HOTE (bottom). The size of the markers is proportional to the number of pairs having that specific combination of ground truth and estimated delay. The dashed line is the identity line *x* = *y*. Black dots far from the identity line correspond to pairs of inferred and ground truth delays that occurred only once. **D** Correlation between ground truth and estimated delays with simulation length. **E** Average error in delay estimation with simulation length. All error bars in the figure are SEM across repetitions of the simulation (*N* = 5)

We could not use information-theoretic measures to infer whether synapses were excitatory or inhibitory as these measures are only positively defined. Therefore, we only examined *XCov* performance in classifying synapses as excitatory or inhibitory. We classified a connection as excitatory and inhibitory based on *XCov*, with positive correlation values assigned as excitatory connections and negative correlation values as inhibitory connections. After 180 min of recording *XCov* could reliably separate excitatory and inhibitory synapses (Fig. 4a). We found that the performance of the classifier increased with simulation time for both the excitatory and the inhibitory class (Fig. 4b).

We also compared how static FC measures performed in inferring ground-truth communication delays. After 180 min of simulation, all static connectivity measures estimated communication delays with a correlation across synapses that was above 0.95 (see Fig. 4c for the relationship between the ground truth delays and those inferred using *XCov*—on the top—and using *HOTE*—on the bottom). The correlation between ground truth and estimated delays grew monotonically with simulation length, with a similar trend for all the measures (Fig. 4d). Nonetheless, *HOTE* estimated the delays more accurately than *XCov* and *TE*. After 180 min of simulation, *HOTE* had an average delay error, measured as the absolute value of the difference between ground truth and inferred delay, of $\left( {0.68 \pm 0.02} \right)$ ms. *XCov* and *T*E showed a systematic error in the delay estimation of approximately 2.5 ms (Fig. 4c, e).

## Relationship between dynamic functional connectivity and the temporal evolution of synaptic weights

Finally, we investigated how the ground truth evolution of the synaptic weights, that is the DSC, related to the measured DFC. We computed DFC using a non-overlapping sliding time window. We first selected a size for the sliding window *T* and then shifted it through the simulated recording in steps of length *T*. We computed DFC only for pairs of neurons that were putatively connected, which we selected as the top 5th percentile of synapses inferred by each measure after 180 min of simulation (Fig. 3e, bottom), and only at the communication delay that we estimated for those synapses (Fig. 4c). Moreover, we computed DFC only for excitatory synapses since the inhibitory ones had a constant synaptic weight in the simulated network (Fig. 2b). We calculated the across-time correlation between DFC and DSC for all synapses to quantify the performance of each FC measure in estimating DSC. To do this, we averaged DSC over windows of width *T*, so that the number of DSC and DFC samples over time were matched.

**Fig. 5:**
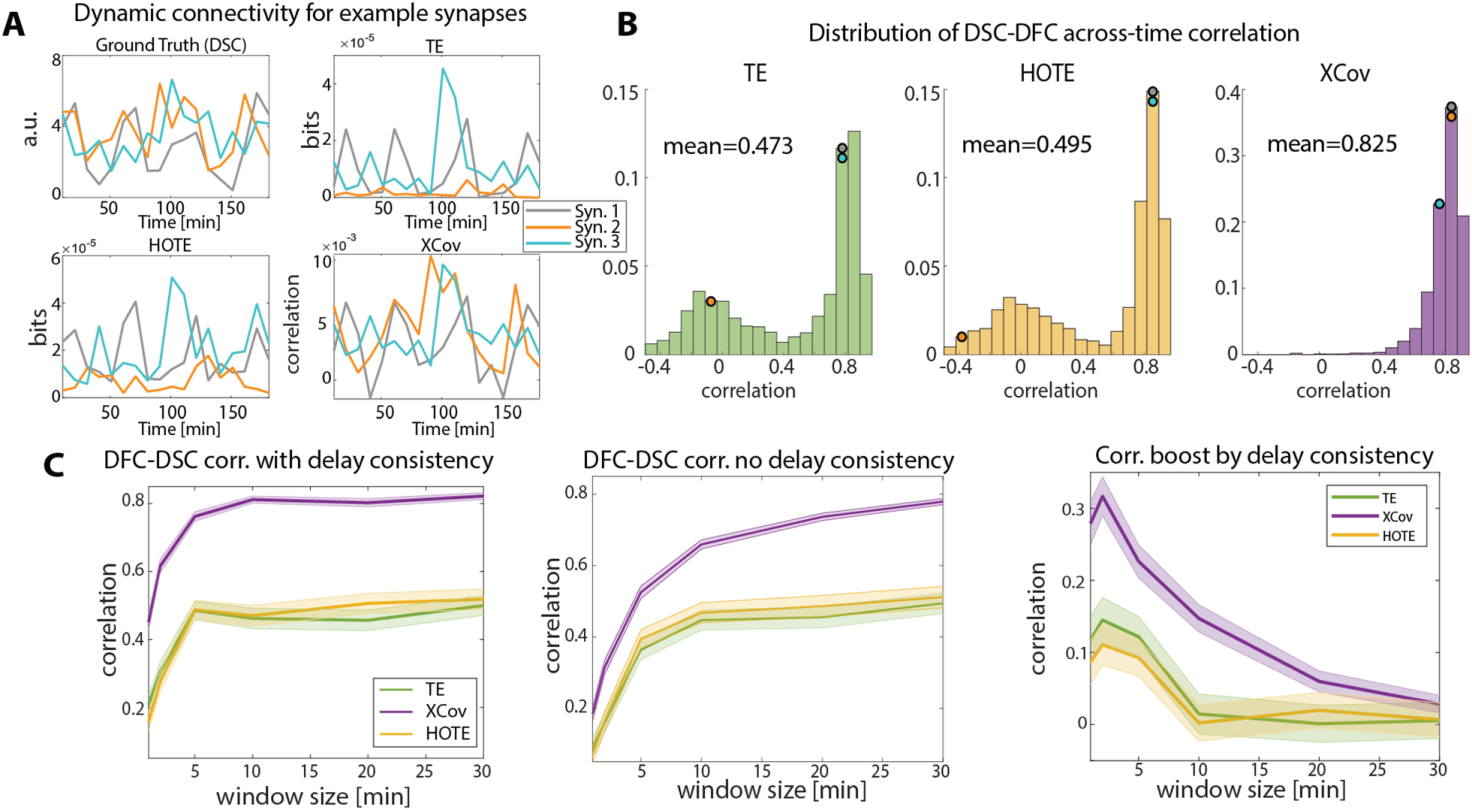
Relationship between dynamic synaptic and functional connectivity. **A** Dynamic connectivity for 3 example synapses, *T* = 10 min. Top left: ground truth dynamics of synaptic weights (DSC). Top right: transfer entropy DFC. Bottom left: HOTE DFC. Bottom right: cross-covariance DFC. **B** Distribution of the across-time correlation coefficients between DSC and DFC, *T* = 10 min. Left: Transfer entropy. Middle: HOTE. Right: cross-covariance. Colored dots show where the synapses in panel A are in the correlation distributions. C Average correlation between DSC and DFC over time for different sizes of the moving window. Shaded areas are SEM across neuron pairs. Left: DFC keeping delay consistency (i.e., measures computed only at previously estimated delay); Middle: DFC without delay consistency; Right: boost in correlation between DFC and DSC when keeping delay consistency (difference between left and middle panels)

In Fig. 5a, we show DSC (top left), DFC computed using TE (top right), *HOTE* (bottom left) and *XCov* (bottom right) for three example synapses and $T = 10\,\min$. While all measures worked reasonably well in tracking how the strength of the grey and the light blue synapses changed over time, *TE* and *HOTE* failed in quantifying the temporal evolution of the orange synapse. We found that, on average, DFC computed via *XCov* correlated with DSC better than DFC computed via *TE* or *HOTE* (Fig. 5b). In particular, while DFC computed via *TE* and *HOTE* had a high temporal correlation with DSC (above 0.7) for the majority of neuron pairs, their distributions showed a large tail of pairs whose correlation between DSC and DFC was distributed around zero (such as the orange one in Fig. 5a). For *XCov*, the number of synapses whose DSC was poorly estimated decreased rapidly with the correlation strength, and the average correlation was 0.82 (Fig. 5b, right). Therefore, DFC computed using *XCov* outperformed DFC obtained from *TE* and *HOTE* in inferring the ground truth changes of synaptic weights over time.

We then studied how the across-time correlation between DSC and DFC depended on the width of the sliding window $T$. Differently from the earlier conference presentation of this work [23], we subsampled the number of time-points in DSC and DFC time series obtained with different *T* to match the number of samples we had for *T* = 30 min. The number of samples in the time series is inversely proportional to *T*, thus a fair comparison of DSC and DFC correlation for different *T* requires the number of samples used to compute correlation to be matched [32]. The correlation between DFC and DSC increased with window size, reaching a plateau around $T = 10\,\min$ (Fig. 5c, left). Below $T = 10\,\min$, the correlation dropped due to the limited sample size used to compute DFC manifesting a tradeoff between the size of the sliding window $T$, which is also the temporal resolution of DFC measures, and the performance in estimating DSC. Nonetheless, the correlation between DSC and DFC was significantly above zero also for sizes of the sliding window similar to the width of the synaptic weights autocorrelation (Fig. 2c). We repeated the same analysis without keeping the delay consistent when computing DFC but simply taking the maximum FC value across delays (between 1 and 50 ms) for each window (Fig. 5c, middle). When not keeping the delay consistent with the one inferred  from the static network analysis, the correlation between DSC and DFC dropped substantially. For sizes of the sliding window lower than $T = 10\,\min$, the advantage of keeping a consistent delay was particularly evident, with a boost in correlation between DSC and DFC computed via *XCov* larger than 0.2 (Fig. 5c, right). This result showed a clear benefit in leveraging estimates of delay derived from entire simulated recordings when inferring DSC from DFC.

## Conclusion

We studied how different measures of static and dynamic functional connectivity measured from simulated spiking activity of a recurrent neural network can be used to infer the fixed and time-varying properties of synapses within the network. This question is relevant as in vivo experiments typically rely on recording spiking activity or other functional measures (such as field potentials) to examine network structure using FC. To infer how changes in FC relate to changes in the underlying synaptic structure of the network requires an understanding of the relationship between the static and dynamic FC measures and the fixed and dynamic synaptic properties of the network. We addressed the problem of inferring synaptic weights and their temporal evolution at the level of simulated recordings with single-neuron cellular resolution. As such, our approach differs from and complements other studies of DFC at the level of mass neural activity [33, 34], which lack the ability to resolve interactions between pairs of individual neurons.

We found that among the considered static FC measures, *XCov*, *HOTE* and, in particular, $O^{{\left( {XCov,HOTE} \right)}}$ outperformed other measures in inferring the presence of synapses. Using cross-covariance as a static FC measure could also reliably classify excitatory and inhibitory synapses, while *HOTE* was the best measure to estimate ground-truth communication delay between neurons. Cross-covariance performed best in inferring DSC, with an across-time correlation above 0.8 between DFC and DSC for sliding window sizes larger than 10 min.

We also found that, when computing DFC, keeping the communication delay consistent with the one obtained from the static network analysis increased the correspondence between DFC and DSC, especially for sliding windows shorter than 10 min. This benefit is likely to arise from the fact that, in situations like those simulated here in which the communication delay is a fixed structural property of the neuron pair over the considered time scales, estimating the delay from long time windows increases the precision of its detection without missing out on capturing possible changes of this parameter. This specifically holds under the assumption that communication delays are constant in the recording period as is the case of our spiking network.

Reliable methods to infer structural properties of neural networks are relevant to several open questions in system neuroscience, ranging from investigating the relationship between structural connectivity and computational properties of neural populations to understanding the physiological mechanisms that control the up- and down-scaling of FC, e.g., how the dynamics of synaptic weights relate to changes in functional connectivity during sleep. Another relevant potential application of such methods concerns the inference of STDP rules from recordings of spiking activity. Many studies support the idea that several STDP rules might coexist in different cells or brain areas [35, 36]. Nonetheless, such theories are complicated to test in vivo due to lack of statistical methodologies to estimate how synaptic weights evolve after STPD-triggering events. The methods presented in this work could potentially be used to infer STDP rules governing network plasticity from in vivo recordings, by estimating how synaptic weights change after the occurrence of pre- and post-synaptic spikes with precise temporal relationships.

The present study has limitations that we plan to address in future works. First of all, it will be important to validate DFC measures on more biologically realistic simulated neural networks with global oscillations, correlated inputs to neurons or global network covariations (which induce FC not related to direct synaptic connections between the neurons [37, 38]), and more heterogeneity in the firing rates and in the average synaptic weights over time. Such effects could act as confounders of the relationship between DFC and DSC or could require refined null hypotheses based on permutation tests to assess the presence of synapses. In the model we also assumed that communication delays between neurons are fixed and no synapses are formed or eliminated over time. The former assumes that the main parameters determining the conductance velocity of action potentials (e.g., axons diameters and myelination) are approximately constant over time scales of a few hours. Experimental finding suggest that this assumption is reasonable, especially in adult mice where the formation of new myelin occurs in the range of weeks [39]. The latter assumption is more delicate since in mice it has been shown that, especially during sleep, dendritic spines can be formed and eliminated within hours [15]. It will be important to investigate how much we can relax these hypotheses while still exploiting the knowledge obtained from static FC measures. Moreover, we plan to test the performance of other bivariate (e.g., Granger Causality) and multivariate measures for estimating DSC. These measures include using Granger Causality estimates based on Generalized Linear Models [40–42] and maximum entropy models [43, 44]. Such multivariate measures could be useful to alleviate the effect of confounders such as common inputs.

Lastly, it will be crucial to apply such methods to data collected from real neural populations and validate, in the first place, the performance of inferring fixed structural connectivity properties from static FC (Figs. 3, 4). A first way to validate the method proposed here is to verify if the static connectivity networks obtained from two long (e.g., >90 min) independent recordings of the same population converge to the same inferred synapses and delays. A second possible validation of the static part of our methodology would be to apply the FC measures to a long recording of a population whose fixed structural properties were reconstructed post-mortem using, e.g., electron microscopy [5, 45]. Such methods typically identify the synapses of neurons whose functional activity was recorded with two-photon calcium imaging rather than with electrophysiology. Given the lower signal-to-noise ratio and temporal resolution of calcium imaging recordings [46], it would be important to first extend and then validate in simulations our proposed methodology to simulated two-photon imaging recordings, rather than simulated electrophysiological recordings as done here.

In conclusion, here we laid down foundations for relating dynamic functional connectivity to the temporal evolution of synaptic weights in spiking neural networks. The results obtained here provide a benchmark for further improving methodologies that infer DSC from DFC.

## Data Availability

The MATLAB source-code used to generate and analyze the data of this study can be downloaded at https://github.com/mcelotto/estimate_synWeigths_from_DFC. The software is released under the MIT license. The code used to compute the TE and HOTE measures is taken from [21] and can be downloaded at https://code.google.com/archive/p/transfer-entropy-toolbox/.

## Abbreviations

STDP: Spike timing-dependent plasticity
FC: Functional connectivity
DFC: Dynamic functional connectivity
DSC: Dynamic synaptic connectivity
XCorr: Cross-correlation
XCov: Cross-covariance
TE: Transfer entropy
HOTE: Higher order transfer entropy
PR: Precision–recall
TP: True positive
FP: False positive
FN: False negative
AUPR: Area under the precision–recall curve
E–E: Excitatory-to-excitatory
E–I: Excitatory-to-inhibitory
I–E: Inhibitory-to-excitatory
I–I: Inhibitory-to-inhibitory
